# *In planta* Protein Interactions of Three Alphacryptoviruses and Three Betacryptoviruses from White Clover, Red Clover and Dill by Bimolecular Fluorescence Complementation Analysis

**DOI:** 10.3390/v5102512

**Published:** 2013-10-09

**Authors:** Till Lesker, Edgar Maiss

**Affiliations:** Department of Phytomedicine, Institute of Horticultural Production Systems, Leibniz Universität Hannover, Herrenhäuser Str. 2, Hannover D-30419, Germany; E-Mail: lesker@ipp.uni-hannover.de

**Keywords:** *Partitiviridae*, *Alphacryptovirus*, *Betacryptovirus*, protein interaction, Bimolecular fluorescence complementation (BiFC)

## Abstract

Plant-infecting viruses of the genera *Alpha- and Betacryptovirus* within the family *Partitiviridae* cause no visible effects on their hosts and are only transmitted by cell division and through gametes. The bipartite dsRNA genome is encoding a RNA-dependent RNA polymerase (RdRp) and a coat protein (CP). Aside from sequence and structural analysis, the investigation of protein interactions is another step towards virus characterization. Therefore, ORFs of two type members *White Clover Cryptic Virus* 1 and 2 (WCCV-1 and WCCV-2), as well as the related viruses from Red Clover and Dill were introduced into a bimolecular fluorescence complementation assay. We showed CP-CP dimerization for all tested viruses with localization for alphacryptoviruses at the nuclear membrane and for betacryptoviruses close to cell walls within the cytoplasm. For CPs of WCCV-1 and WCCV-2, deletion mutants were created to determine internal interaction sites. Moreover, RdRp self-interaction was found for all viruses, whereas CP-RdRp interactions were only detectable for the alphacryptoviruses. An intra-genus test of CPs was successful in various virus combinations, whereas an inter-genus interaction of WCCV-1CP and WCCV-2CP was absent. This is the first report of *in vivo* protein interactions of members in the family *Partitiviridae*, indicating distinct features of the alpha- and betacryptoviruses.

## 1. Introduction

Cryptic viruses, widespread in mono- and dicotyledonous plant species, are currently classified in the genera *Alpha*- and *Betacryptovirus* of the family *Partitiviridae* [1,2]. Additionally, the family contains the genera *Partitivirus* and *Cryspovirus*, which include viruses infecting fungi and protozoa, respectively [2,3]. The genome of cryptic viruses is composed of two monocistronic dsRNA segments of approximately 1.5 to 2.5 kbp in size. While the larger segment encodes a putative RNA-dependent RNA polymerase (RdRp), the smaller one encodes the coat protein (CP). Both dsRNA molecules are individually encapsidated in non-enveloped isometric particles measuring 30–40 nm in diameter [2,4,5]. There are no known natural vectors of plant-infecting cryptic viruses, and they are not transmitted by mechanical means or grafting. Nevertheless, a very high rate of transmission by the gametes is found, nearly 100%, if both parents are infected [6]. Cryptic viruses do not encode proteins with homology to so far known movement proteins of other viruses. Hence their transmission occurs in a passive way by cell division, thereby also infecting seed and pollen [5]. There seems to be a good adaption of cryptic viruses to their hosts, reaching only a low virus titer, persisting for years in tissue culture and withstanding thermotherapy [4]. No visible symptoms are caused by cryptic viruses, and apparently they do not lead to drastic impact on quality and yield in crop plants. Although economic losses in their host plants are not obvious, they can be responsible for misleading results in diagnostic approaches based on RNA detection [4,7]. Plant viruses of the family *Partitiviridae* frequently occur in various species, often in mixed infections with different cryptic viruses and other kinds of dsRNA viruses, such as endornaviruses [8] and viruses similar to *Southern tomato virus* [9,10].

First studies dealing with cryptic viruses were done in the early 1980’s, followed by the first description of their genome structure and particle sizes [4]. Various attempts of virus transmission were made but only an exclusive transmission by seeds and pollen was found. The relationship to mycoviruses was proven by several serological investigations; based on these findings together with particle and genome sizes the classification into the genera *Alphacryptovirus* and *Betacryptovirus* was established. RdRp polymerase activity linked with virus particles was confirmed by enzyme assays [11]. The first viral sequence became available for *Beet cryptic virus 3* [12]. The first complete sequence of an alphacryptovirus, namely *White clover cryptic virus 1* (WCCV-1) was published by Boccardo in 2005 [13], the first betacryptovirus *White clover cryptic virus 2* (WCCV-2) was determined in 2013 [14]. Phylogenetic analyses revealed further subdivision of the genus *Alphacryptovirus* in two clusters and a relationship between herbal and fungal viruses in the family *Partitiviridae* was shown [5,14]. Several studies suggest a viral influence on its host. For example, dsRNA patterns were linked to yellow edge symptoms in radish [15]. In addition, an artificial expression of the WCCV-1CP gene in *Lotus japonicus* influenced the growth of the roots [16]. However, other studies in crop plants were not able to demonstrate any symptoms despite a virus infection or significant impact on yield [5]. In some cases an increase of dsRNA concentration has been observed when an additional plant virus was present together with a cryptic virus [4].

A cryptic virus with a dsRNA genome, but also any other RNA containing virus using dsRNA as a replication intermediate, faces a problem during its replication cycle. Plants natural defense mechanisms generally recognize dsRNA, which is subsequently degraded. RNA viruses have evolved special proteins—suppressors of silencing—to protect themselves in various ways from RNA degradation [17]. Cryptic viruses do not have such kinds of proteins, so they have to hide their dsRNA from the plants natural defense. It is assumed that the dsRNA only occurs in the virus particle itself and here serves as a template for the also encapsidated RdRp [4,5]. The transcribed single-stranded RNA passes from the particle through pores into the cytoplasm, where CP and RdRp are translated [18]. During particle assembly, RNA and RdRp are packaged by protein-protein and protein-RNA interaction together with the CP. Only inside the assembled particle does the RdRp switch to an active mode and start to synthesize new dsRNA [18].

Recent X-ray diffraction studies focused on the structural analyses of virus particles. A 3D model was established for *Penicillium stoloniferum virus F* (PSV-F) a member of the genus *Partitivirus*, which is closely related to plant infecting alpha- and betacryptoviruses. The particle composition follows a T = 1 symmetry consisting of 120 subunits [19]. Furthermore, pores were found suitable for mRNA transfer; however, RdRp was not localized in particles [20]. A biological characterization of cryptic viruses is difficult because of their features, like a limited transmission. This also applies to the establishment of reverse genetic systems due to the dsRNA nature of these viruses.

After genetic studies concerning plant cryptic viruses [6,14] identification and investigation of protein–protein interactions present a further step in understanding the virus biology of the alpha- and betacryptoviruses. Several methods were established to identify and characterize protein-protein interactions. Besides different *in vitro* methods [21], the yeast two-hybrid (YTH) system [22] is the most popular *in vivo* method to detect protein interactions. However, this system relies on the yeast nucleus under artificial conditions. Protein interactions requiring biologically relevant modifications or a specific subcellular localization are not detectable [23]. Therefore, bimolecular fluorescence complementation (BiFC) analysis was developed and became a powerful alternative for studying protein-protein interactions [24,25]. The two proteins of interest (POI) are fused to the non-fluorescent N-terminal or C-terminal fragment of a fluorescent protein. If the POI interact with each other, both parts of the reporter become reconstituted and fluorescence can be detected. Significant advantages of this system are the high specificity and great stability of the reconstituted chromophore complex and its intrinsic fluorescence under natural conditions. Furthermore, it is possible to localize the protein interactions in the cell.

In this study, an optimized BiFC-system [26] was used to investigate for the first-time protein interactions of viruses belonging to the family *Partitiviridae* *in planta*. The aim was to verify expected and hypothesized protein interactions. Firstly, we focused on the CP dimerization, which is the starting point of virus assembly. Sixty of these dimers are building the particle structure of *Partitiviridae* with a T = 1 symmetry, whereas no additional viral components are needed for this domain swapping. Furthermore, we hypothesized an interaction of CP and RdRp. This interaction is proposed for the last steps of the virus assembly to introduce the RdRp in the particle and to activate the transcription [5]. Additionally, self-interaction of the RdRp was tested. For clarification of functional relationships among the cryptic viruses and to establish negative controls for the BiFC-system the CP and RdRp of one virus were tested *versus* proteins of two other virus members of the same genus (interspecies interactions). Moreover, an intergenus interaction with the CPs of WCCV-1 and WCCV-2 was performed. Additionally, we used deletion mutants to narrow down the part involved in the CP-CP interaction of the two type members of alphacryptovirus, WCCV-1 and betacryptovirus, WCCV-2, respectively.

## 2. Results and Discussion

Due to the formerly described infection cycle of the cryptic viruses in plants, different protein-protein and protein-RNA interaction could be expected. The primary domain shaping of CP proteins to dimers forms the basis of the final capsid structure. Multiple interaction sites were found by structural analyses [20,27], so a CP self-interaction could be expected in different permutations, as well as in distinct deletion mutants. Moreover, due to the fundamental similarities, interactions between CPs from viruses found in related host plants (intra genus) are most likely. The only other encoded protein, the RdRp, has to be packaged into the particle, where it is assumed to recognize higher CP- or RNA-structures to start transcription and the synthesis of the dsRNA genome [5]. The viral genome within the particle is hidden from the plant defense mechanisms centered on the recognition of dsRNA.

Another important step in the virus life cycle is to ensure the passive transport of cryptic viruses during cell division, especially to the gametes. Due to the lack of movement proteins for active transport via plasmodesmata, the cryptic viruses had to develop mechanisms to establish in meristem cells, which enable them to withstand thermotherapy [3]. An interaction and *in planta* localization approach could be the first step to provide more hints to understand the “cryptic strategy”.

An optimized BiFC system was used to elucidate protein interactions of six different cryptic viruses from the genera *Alphacryptovirus* and *Betacryptovirus*. For this purpose, the type members WCCV-1 and WCCV-2 from *Trifolium repens* [28] and two closely related cryptic viruses from *Trifolium pratense*, namely Red clover cryptic virus 1 and *Red clover cryptic virus 2* [29] were used. In addition, the more distantly related Dill cryptic virus 1 and Dill cryptic virus 2 [14] from *Anethum graveolens* of the family *Apiaceae* are also included in the study.

### 2.1. Establishment of Internal Controls

**Figure 1:**
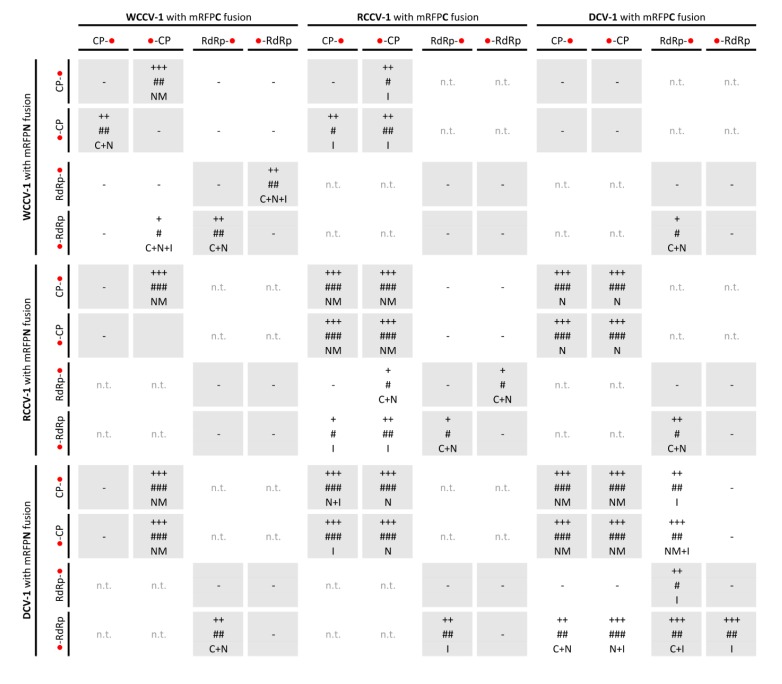
Interactions of the RNA-dependent RNA polymerase (RdRp) and coat protein (CP) of alphacryptoviruses WCCV-1, RCCV-1 and DCV-1. Grey shaded areas indicate self-interactions of CP and/or RdRp. Symbols: “−”: no fluorescence; “n.t.”: not tested; “+++”/“++”/“+”: for strong/medium/low fluorescence signals; “###”/“##”/“#”: almost all/mean number of/only a few cells detected with fluorescence; capital letters indicate localization of fluorescence in the cell: “C”: cytoplasm, “I”: inclusions in the cytoplasm, “N”: nucleus, “NM”: nuclear membrane. Bimolecular fluorescence complementation (BiFC) constructs are represented in the vertical line with BiFC3 (CP-mRFPN): “CP-●”, BiFC1 (mRFPN-CP): “●-CP”; or BiFC3 (RdRp-mRFPN): “RdRp-●”, BiFC1 (mRFPN-RdRp)”: ●-RdRp” and in the horizontal line with BiFC4 (CP-mRFPC): “CP-●”, BiFC2 (mRFPC-CP): “●-CP” or BiFC4 (RdRp-mRFPC): “RdRp-●”, BiFC2(mRFPC-RdRp): “●-RdRp”.

**Figure 2:**
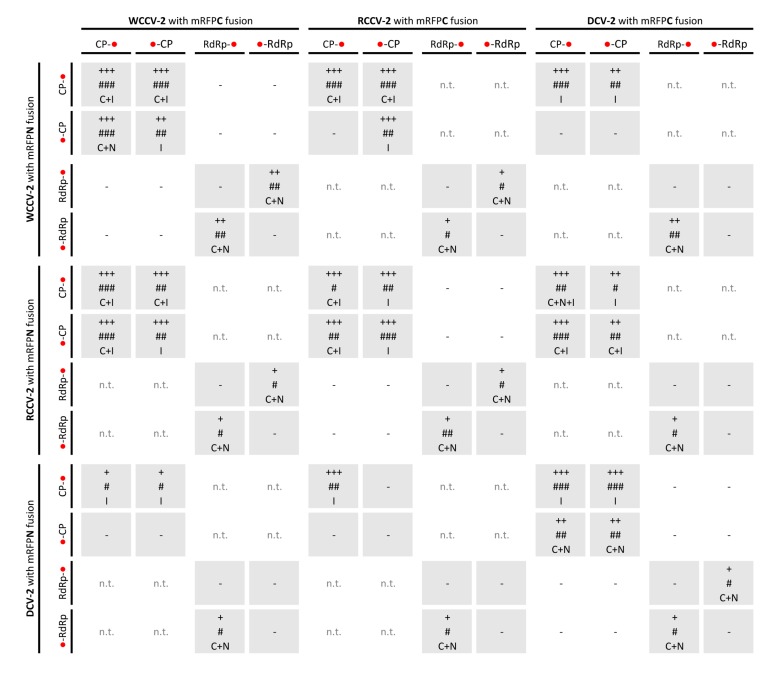
Interactions of RdRp and CP of betacryptoviruses WCCV-2, RCCV-2 and DCV-2. Grey shaded areas indicate self-interactions of CP and/or RdRp. Symbols: “−”: no fluorescence; “n.t.”: not tested; “+++”/“++”/“+”: for strong/medium/low fluorescence signals; “###”/“##”/“#”: almost all/mean number of/only a few cells detected with fluorescence; capital letters indicate localization of fluorescence in the cell: “C”: cytoplasm, “I”: inclusions in the cytoplasm, “N”: nucleus, “NM”: nuclear membrane. BiFC constructs are represented in the vertical line with BiFC3 (CP-mRFPN): “CP-●”, BiFC1 (mRFPN-CP): “●-CP”; or BiFC3 (RdRp-mRFPN): “RdRp-●”, BiFC1 (mRFPN-RdRp): “●-RdRp” and in the horizontal line with BiFC4 (CP-mRFPC): “CP-●”, BiFC2 (mRFPC-CP): “●-CP” or BiFC4 (RdRp-mRFPC): “RdRp-●”, BiFC2(mRFPC-RdRp): “●-RdRp”.

Initially, the Plum pox virus coat protein and deletion mutants thereof served as positive and negative controls, *i.e.*, to verify protein-protein interactions detected by the BiFC system. The development of controls with proteins of cryptic viruses is limited, because these viruses encode only two proteins, which largely reduces the number of possible interaction partners. To circumvent this drawback, proteins of closely and distantly related cryptic viruses from two different genera were used in this study to broaden up the spectrum of potential interaction partners. To ensure the association of the monomeric red fluorescent protein (mRFP) fragments each CP and RdRp protein was fused to the N- as well as the C-terminal fragment. This allowed a screening of multiple combinations of fusion proteins for fluorescence complementation in all permutations (Figure 1 and Figure 2). A total of four different BiFC binary vectors (BiFC 1–4) resulted, which carry the RdRp and CP genes, respectively, of distinctive cryptic viruses. Finally, for each CP and RdRp self-interaction four constructs and for the RdRp-CP interaction eight-constructs were available to test the interactions.

In at least one combination of each construct (Virus–CP/RdRp–BiFC-Vector 1–4) an interaction was found (Figure 1 and Figure 2). This indicates a correct translation of fusion proteins, because in case of binary vectors BiFC3 and BiFC4 the GOI was fused upstream to the reporter gene. In the BiFC1 and BiFC2 vectors identical GOI-fragments from BiFC3 and BiFC4 were used, and the final constructs were verified by restriction enzyme digest and sequencing. Therefore, the different permutation and cross species tests of each construct served also as either additional positive or negative control. In case of BiFC2 mRFPC-RCCV-1CP only positive interactions with all test partners were identified, whereas with all other constructs, at least one negative interaction was determined. Thereby, additional control measurements with BiFC2 mRFPC-RCCV1CP were performed to exclude false-positive results, e.g., testing without any interacting partner, which reveals no fluorescence (data not shown). In addition, different localizations with BiFC2 mRFPC-RCCV1CP were found in several interactions, indicating the correct and specific determination of interactions and no general and unspecific interaction of the test partners.

Interestingly, in several cases interactions were not found in all kinds of permutations. Especially RdRp self-interactions were only found in combinations when the RdRp was fused N-terminal as well as C-terminal to the mRFP with the BiFC2/3 vectors or *vice versa* with the BiFC1/4 vectors (Figure 1 and Figure 2). This indicates that testing of all permutations might be beneficial in case of all BiFC systems. If only one permutation is tested with a negative result, all other permutations should also be tested to avoid the oversight of possible interacting partners. This applies to studies on the localization of interactions, too. In case of self-interactions of RCCV-1CP and DCV-1CP an association with the nuclear membrane was evident (Figure 1) with all BiFC combinations. However, the same expected localization of WCCV-1CP was found only in one permutation (BiFC2/3).

### 2.2. CP Dimer Formation

The particles of the *Partitiviridae* are composed of 120 CP subunits forming 60 dimers, which corresponds to a T = 1 symmetry [2]. For virus assembly of cryptic viruses, interactions between CP subunits, the RdRp and RNA are necessary. A certain degree of self-assembly without any other viral element occurs for the CP subunits of viruses. Furthermore, even entire particles without encapsidated RNA were found in case of isometric viruses [30,31]. CP dimers act as starting points for the assembly process [20].

An interaction of the CP was detected for all alpha- and betacryptoviruses (Table 1 and Table 2). Detection of WCCV-1 CP-CP interaction depended on the localization of the fused protein in relation to the mRFP-fragment as described above. Furthermore, differences in the number of cells showing fluorescence and also in the intensity of the fluorescence were observed. A strong fluorescence signal was found in the majority of epidermal cells within the analyzed leaf regions (Figure 1).

**Table 1:** Schematic overview of the tested alphacryptovirus WCCV-1CP deletion mutants; “−” no interaction visible; “+++”/“++”/“+”: for strong/medium/low fluorescence signal; “###”/“##”/“#”: almost all /mean number of /only few cells detected with fluorescence; capital letters for localization in the cell: “C”: cytoplasm, “N”: nucleus, “NM”: nuclear membrane.

	BiFC2: F-mRFPC			Full	F1	F2	F3	F1/2	F2/3	F1/3
BiFC3: mRFPN-F										
				+++	+		+++	+	++	+++
**Full**	F1	F2	F3	###	#		###	#	##	###
				NM	C + N	–	NM	C + N	NM	NM
**F1**	F1			–	–	–	–	–	–	–
	
								++	–	
**F2**		F2		–	–	–	–	##		–
	
								C + N		
**F3**			F3	–	–	–	–	–	–	–
	
**F1/2**	F1	F2		–	–	–	–	–	–	–

								++		
**F2/3**		F2	F3	–	–	–	–	##	–	–

								C+N		
**F1/3**	F1		F3	–	–	–	–	–	–	–


**Table 2:** Schematic overview of the tested betacryptovirus WCCV-2CP deletion mutants; “−”no interaction visible; “+++”/“++”/“+”: for strong/medium/low fluorescence signal; “###”/”##”/”#”: almost all /mean number of /only few cells detected with fluorescence; capital letters for localization in the cell: “C”: cytoplasm, “I”: inclusion in cytoplasm.

	BiFC4: mRFPC-F			Full	F1	F2	F3	F1/2	F2/3	F1/3
BiFC3: mRFPN-F										
				+++		+++	+	++	+	
**Full**	F1	F2	F3	###	-	###	#	##	#	-
				C + I		I	I	I	I	
**F1**	F1			-	-	-	-	-	-	-
	
						+++		++	++	
**F2**		F2		-	-	###	-	#	##	-
	
						I		I	I	
						++				
**F3**			F3	-	-	##	-	-	-	-
	
						I				
				++		++		++	++	
**F1/2**	F1	F2		##	-	#	-	#	##	-

				C + I		I		I	I	
						++				
**F2/3**		F2	F3	-	-	##	-	-	-	-

						I				
**F1/3**	F1		F3	-	-	-	-	-	-	-


**Figure 3:**
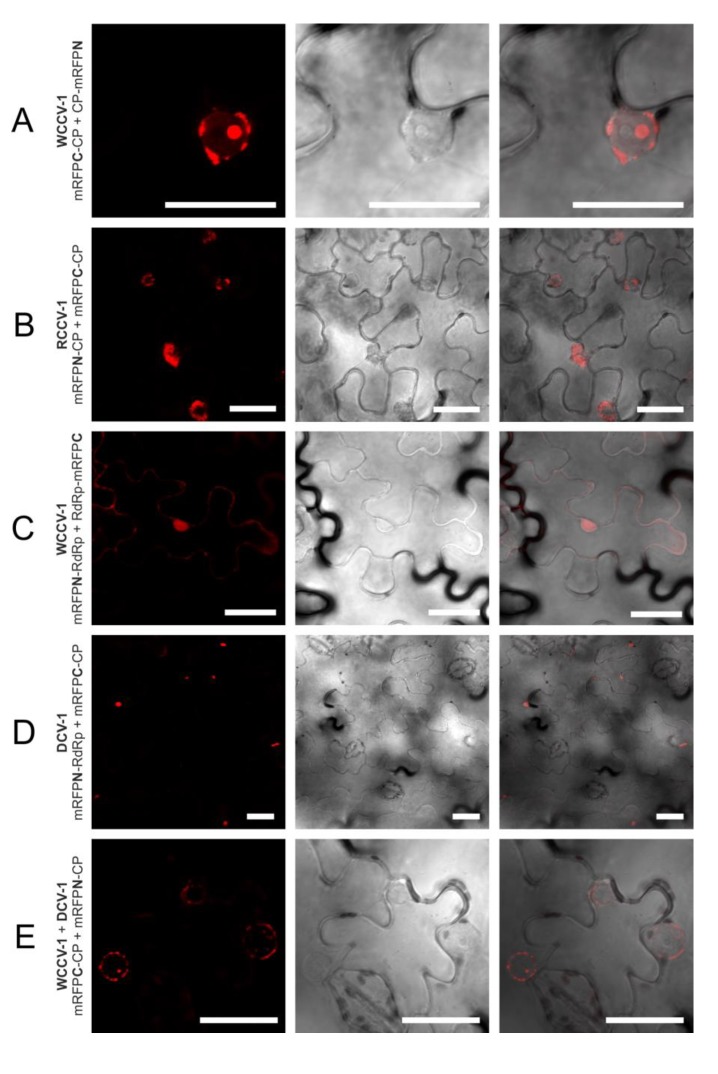
Selected interactions of proteins of alphacryptoviruses. BiFC of mRFP in *N. benthamiana* epidermal cells at three days p.i. CLSM images for the mRFP fluorescence, the transmitted light mode of chlorophyll and merged pictures with the transmitted light mode of cells. Bars, 30 µM.

The CP interaction of viruses of the same genus in plant cells was localized in as similar manner, but differs in the alpha- and betacryptoviruses. Concerning the alphacryptoviruses all three tested viruses showed CP homo-dimer formation. A localization of CP-CP homo-dimers at the membrane surrounding the nucleus was visualized (Figure 1; Figure 3A,B), in regard to RCCV-1 and DCV-1 even in all four permutations. Prominent deposits could be found associated with the outer membrane without fluorescence inside the nucleus. In addition, CP-CP hetero-dimers were detected between WCCV-1, RCCV-1 and DCV-1 (Figure 3E), respectively, but again not in all permutations.

**Figure 4:**
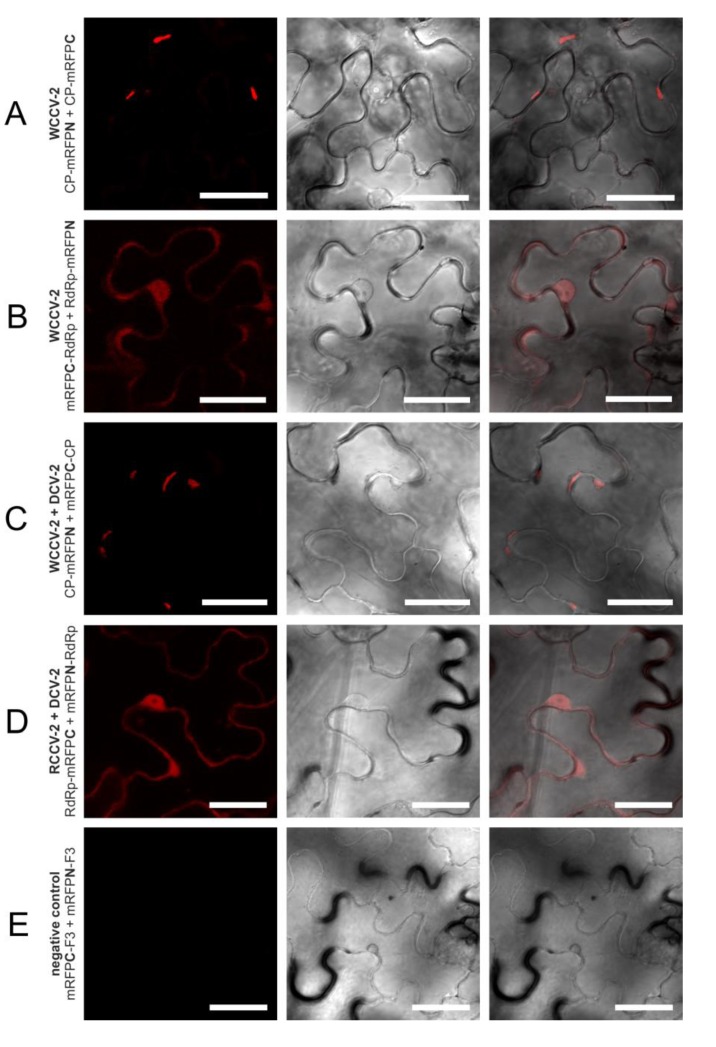
Selected interactions of proteins of betacryptoviruses. BiFC of mRFP in *N. benthamiana* epidermal cells at three days p.i. CLSM images for the mRFP fluorescence, the transmitted light mode of chlorophyll and merged pictures with the transmitted light mode of cells. Bars, 30 µM.

In a similar way, all intragenus permutations of the CP of the betacryptoviruses were tested. A distinct localization for CP interactions of viruses from the genus Betacryptovirus was absent (Figure 2). In contrast to the alphacryptoviruses protein-protein interactions were mainly detected in marginalized deposits in the cytoplasm close to the cell wall (Figure 4A). These inclusion bodies in the cytoplasm can consist of biologically inactive proteins. However, CP-CP interactions were also detected by fluorescence in the cytoplasm and the nucleus for WCCV-2 and DCV-2. Moreover, CP-CP interspecies interactions were as well detected between WCCV-2, RCCV-2 and DCV-2 (Figure 4C), respectively, but similar to alphacryptoviruses not in all permutations.

Lastly, no intergenus interaction between the CP of alphacryptovirus WCCV-1 and the Betacryptovirus WCCV-2 was detected independent of BiFC permutations (results not shown).

However, except for WCCV-1 a CP interaction was demonstrated in all permutations of the tested viruses, which indicates interacting domains or areas independent of free N- and C-termini of the CPs. The 40 N-terminal amino acids of Partitivirus CPs were not involved in the structure of the particle resolved by 3D structure analyses [20,27]. Probably, they are located at the inside of the virus particle and ensure the arrangement of the dsRNA within the particle [18] or they are located at the surface of the virion.

A more precise localization in cell compartments could be reached with other techniques like immune labeling electron microscopy in the host plants or *in situ* hybridization. However, the distinctive location of primary virus assembly sites showed in this study may indicate that the viruses of the genera *Alphacryptovirus* and *Betacryptovirus* use different compartments to co-exist in one cell.

### 2.3. Localization of Protein Interaction Sites in the CPs

The putative interaction domain within the WCCV-1CP and WCCV-2CP was approximated by dividing the coding frame into three parts. The fragments vary from 150 to 273 amino acids, so that protein structures should be formed. However, possible secondary structures were not taken into consideration for the choice of the selected regions. Moreover, each potential interaction of different fragments was tested with only two fusion permutations, resulting in a limited degree of freedom for protein adjustments. The particle structure, as outlined above, implied multiple interactions within a single CP for dimer formation. In addition, protein regions are known that are probably not at the surface of viral particles [20,27] and more likely bind RNA inside the particle [18].

To narrow down the interacting domains in the CP of WCCV-1 and WCCV-2, six different deletion mutants were created (Table 1). We obtained only a few interactions for the alphacryptovirus WCCV-1CP mutants, similar to the findings for the full-length CP permutation tests. In the used BiFC2/3 permutation (mRFPC-F/F-mRFPN) only seven interactions out of 48 possible combinations tested positively. The full-length CP in the BiFC3 vector was interacted with all other BiFC2 (mRFPC-CP) fragments except for BiFC2-F2. Additionally, we also detected interactions for BiFC2-F1/2 with BiFC3-F2 and BiFC3-F2/3. Furthermore, the localization of the observed fluorescence in the BiFC2-F1 and BiFC2-F1/2 combination changed from the nuclear membrane to the cytoplasm and nucleus compared to the interaction of the full-CP used as a positive control (Table 1).

The orientation of the fusion in respect to the reporter part seems to be critical for the dimerization. The association with the C-termini of the full-length CP resulted in five detected interactions with CP fragments, whereas the opposite direction did not. It is particularly interesting to note that only if both partners include the F3-part the fluorescence was located on the nuclear membrane. This might be an indication that the C-terminus is involved in the protein localization perhaps it provides its own signal peptide sequence for the protein targeting.

A similar approach was performed for the Betacryptovirus WCCV-2 (BiFC3/4 permutation; F-mRFPN-F/F-mRFPC; Table 2). Overall, thirteen interactions out of 48 possible combinations were identified. Fluorescence was mainly located in inclusions within the cytoplasm of the plant cells. Most interactions were detected for mutants still including the F2 part. In contrast, no interactions were observed in any combination with F1 and F1/3 fragments. Furthermore, BiFC3-F3 mutant interacts with BiFC4-F2 and BiFC4-F3 with the BiFC3 full length CP, but F3 in BiFC3 and BiFC4 did not interact with itself (Figure 4E). Conversely, almost all positive combinations of interaction required the F2 part in both partners, and additionally, for the F2 fragment an interaction with the F3-mRFPN fusion was shown. This furthermore indicates that the middle part–F2–of the WCCV-2 protein is particularly important for primary dimerization and probably also for the forming of inclusions within the cytoplasm.

### 2.4. RdRp Dimerization

The RdRp of *Partitiviridae* is located within the virus particle and produces transcripts of the dsRNA genome. The transcripts are delivered through pores of the particle into the cytoplasm [2]. The tested viruses have only one dsRNA per particle and accordingly just one RdRp molecule will be packaged [30,31]. Therefore, RdRp self-interaction seems not necessary. However, in all alpha- and betacryptoviruses a potential RdRp interaction was found, almost always in the BiFC1/4 permutation (mRFPN-RdRp with RdRp-mRFPC) and BiFC2/3 (mRFPC-RdRp with RdRp-mRFPN) combination, in which the RdRp was fused N- and C-terminal to the mRFP.

The fluorescence was predominantly observed in the cytoplasm and the nucleus as shown for WCCV-1 (Figure 3C) and WCCV-2 (Figure 4B). For DCV-1, a RdRp interaction was detected with all permutations except BiFC1/4 (mRFPN-RdRp with RdRp-mRFPC). The fluorescence was observed equally distributed throughout the cytoplasm but also in inclusions within the cytoplasm. Additionally, an RdRp interaction of RCCV-2 and DCV-2 was shown resembling the homologous interaction (Figure 4D).

However, RdRp interactions were less frequent than CP interactions. Overall, also the fluorescence intensity and frequency of cells showing fluorescence was lower compared to the CP interactions (Figure 1 and Figure 2), indicating for a weak and fragile self-interaction. Furthermore, a close proximity of overexpression, aggregation and mis-localization of RdRp proteins may contribute to the interaction determined by the BiFC-system. Dimer formation of RdRps was also described for other virus families [32]. However, these clearly differ in their replication cycle from *Partitiviridae*. Former publications gave no evidence of a RdRp self-interaction. Therefore, further analyses like yeast two hybrid analyses concerning the weak but clearly detectable RdRp self-interaction might confirm the results.

### 2.5. CP-RdRp Interactions

During particle assembly of cryptic viruses, RNA and RdRp have to be assembled with the CP [2]. 3D structural analyses have shown pores within the particle that might support the transfer of newly synthesized RNA by RdRp from the particle [20,27]. These pores are small but flexible, so that an interaction between RNA and/or RdRp resulting in a transfer of RNA can be supposed [18,20]. RdRp could not be shown in structural analyses [20]. It is postulated that RdRp is not covalently attached to the inside of the particle [2]. Nevertheless, the RdRp should be localized within the particle to transcribe and to convert ssRNA into dsRNA [5].

An interaction of CP and RdRp has been observed in at least one permutation of all alphacryptoviruses. Several permutations showed a medium fluorescence in a few cells. In regard to WCCV-1 only the BiFC1/2 combination (mRFPN-RdRp with mRFPC-CP) delivered a positive signal (Table 1). Three interactions were found in fusions of RdRp with mRFPN and CP with mRFPC in the closely related RCCV-1 but these interactions were not detectable in the opposite orientation of the tested proteins. In contrast, we observed plenty of intensely fluorescent cells in case of DCV-1, in all permutations of the C-terminal RdRp fusions (Figure 1). The localization of the proteins within the cell was not homogeneous; positive fluorescence signals were mainly found in inclusions in the cytoplasm (Figure 1 and Figure 3D), in some permutations in the cytoplasm itself and in the nucleus and nuclear membrane.

So the localization, in case of WCCV-1 and RCCV-1, clearly differs from RdRp and CP self-interactions, where greater deposits in the cells were missing. A localization of CP-RdRp interaction at or near the outer nuclear membrane as described for the primary dimer fusion was found in one permutation only. This might be an indication for a later CP-RdRp interaction step within the framework of virus assembly in the cytoplasm.

In contrast to alphacryptoviruses no interaction between CP and RdRp was found in the three viruses of the genus *Betacryptovirus* (see Figure 2). One reason could be that the CP-RdRp interaction is weaker in manifestation and therefore, harder to verify with the used BiFC-system. The particles differ from those of the alphacryptovirus in particle size—38 *vs.* 30 nm—and the presence of prominent subunits on the particle surface [5]. Other factors like higher structures of CPs or the presence of full‑length RNA may be a prerequisite and essential for CP-RdRp interaction. Concerning these points further analyses like trimolecular fluorescence complementation analysis [33] might be helpful.

### 2.6. Cross Species Interaction of CP and RdRp

Cryptic viruses are interesting regarding their evolutionary relationship to one another, because a horizontal transmission of those viruses via vectors is not known [2]. However, there is a high sequence homology in these viruses, even though they occur in different plant families [5]. From the phylogenetic point of view, a horizontal transmission with a vector is more likely than a coevolution between the virus and the host before primeval times [14]. Besides using interspecies tests as internal controls it was interesting to find out if protein interactions can also be established among the viruses within one genus.

We tested the CP and RdRp hetero dimerization of related viruses within one genus from white clover and red clover, furthermore, of more distantly related viruses from dill. CP dimers were detected between all viruses within one genus (Figure 1 and Figure 2). Concerning the alphacryptoviruses a strong fluorescence was found in almost all cells, localized in analogy to the already described CP dimers in the membrane of the nucleus (Figure 3E). Nevertheless, also a different localization in the nucleus for the dimers RCCV-1 CP and DCV-1 CP was visible. Moreover, eight permutations with WCCV-1 revealed no interaction, and in another three permutations only a few cells were detected with low fluorescence signals from inclusions located in the cytoplasm.

In regard to the betacryptoviruses a similar localization for CP hetero-dimers were observed and overall fewer combinations showed a positive signal with a lower number of cells and fewer intensities of fluorescence, especially in combination with the more distantly related DCV-2. Interactions occurred in all tested virus combinations. In particular, it was noticed that interactions were not found with all permutations, compared to self-interactions, the fluorescence was weaker and the localization changed. This was also the case for the RdRp hetero dimerization detected within the genus *Alphacryptovirus*for DCV-1 with WCCV-1 and RCCV-1, respectively, in the BiFC1/4 (mRFPN-RdRp with RdRp-mRFPC) combination as already described above, but not between WCCV-1 and RCCV-1. In addition, a dimerization for all RdRps of the betacryptoviruses was observed.

However, in case of WCCV-1 only a few permutations were found to react positive, which could indicate that RCCV-1 and DCV-1 with much more interactions could be better analyzed in our BiFC system in *N. benthamiana*. Furthermore, an imperfect assembly of virus CPs might cause a malfunction in the further localization. In case of heterologous tests CP subunits of the same virus might preferentially interact, thereby not leading to a fluorescence signal, because of missing one reporter part. Heterologous protein interactions can also induce different localizations like the occurrence of deposits within the cytoplasm in case of the alphacryptovirus. Concerning this, further data are needed for the precise localization and description of steps involved in the virus replication cycle.

## 3. Experimental Section

### 3.1. Construction of the Expression Plasmids for BiFC

The pCB:GOI-mRFPN (BiFC 1), pCB:GOI-mRFPC (BiFC 2), pCB : mRFPN-GOI (BiFC 3) and pCB:mRFPC-GOI (BiFC 4) expression plasmids were generated as described by Zilian and Maiss (2011) [26].

### 3.2. Construction of the Plasmids for Full-Length Protein Interaction

The coding sequence of the CP or RdRp, respectively of WCCV-1, WCCV-2, RCCV-1, RCCV-2, DCV-1, DCV-2 were RT-PCR-amplified using dsRNA preparations from White Clover, Red Clover and Dill with RevertAid Premium Reverse Transcriptase and Phusion Flash Master Mix (Thermo Scientific) as described previously [14]. New sequences are stored in GenBank under accession numbers: RCCV-1RdRp: KF484724, RCCV-1CP: KF484725, DCV-1RdRp: KF484726 and DCV-1CP: KF484727. Fragments were generated by using primers, which include specific restriction endonuclease sites (*Bam*HI or *Bgl*II and *Sal*I or *Xho*I) for cloning into the BiFC vectors (Appendix Table A1). Fragments were first cloned into pJET1.2 (Thermo Scientific) according to the manufacturer’s protocol, digested with the appropriate restriction enzyme and ligated into the binary BiFC-plasmids, which were digested with *Bam*HI/*Sal*I or were cloned by a Gibson Assembly (New England Biolabs) approach [34]. The sequences were verified by sequencing and restriction enzyme digests.

### 3.3. Construction of the Plasmids for Deletion Mutants of WCCV-1CP and WCCV-2CP

The open reading frame of each CP was divided into six fragments by PCR mutagenesis using Phusion Flash DNA polymerase (Thermo Scientific). The F1, F3, F1/2, F2/3 and F1/3 fragments of WCCV-1CP, encoding aa 1–150, 151–338 and 339–487, respectively were generated using the BiFC2:mRFPC-WCCV-1CP and BiFC3:WCCV-1CP-mRFPN vectors as templates. The same fragments of WCCV-2CP, using aa 1–200, 201–473 and 474–673 were created from the BiFC3: WCCV-2CP-mRFPN and BiFC4: WCCV-2CP-mRFPNC vectors.

### 3.4. Transient Protein Expression in N. benthamiana Leaf Epidermal Cells and CLSM

The BiFC plasmids and pCB:p35TBSVp19, encoding the TBSV p19 protein as a suppressor of gene silencing, were used for the electroporation into *A. tumefaciens* strain GV2260 [35]. Agrobacteria cultures harbouring the plasmids were prepared for infiltration according to Zilian & Maiss (2011) [26]. The infiltration of young leaves of *N. benthamiana* plants 4 to 5 weeks old was performed by using *A. tumefaciens* mixtures containing the BiFC1-4 plasmids and pCB:p35TBSVp19 binary plasmid. All infiltrated plants were incubated at room temperature for 3 days. Discs of infiltrated *N. benthamiana* leaves were investigated with a Leica TCS SP2 confocal laser scanning microscope. The excitation at 543 nm of the mRFP domain was performed by using the green neon laser. The emitted light was captured at 600–610 nm, thus creating consistent-recording conditions. Visualization of the chlorophyll autofluorescence was made by excitation at 488 nm with the argon/crypton laser and subsequent fluorescence detection at 690–740 nm. Digital capture and processing of the images were performed by using the Leica confocal software.

## 4. Conclusions

Our results revealed various differences in protein interactions between alpha- and betacryptoviruses, which are not only caused by different protein and particle sizes. As already described, betacryptoviruses differ from alphacryptovirus in terms of the presence of prominent arches on the virus particle surface [4]. For the betacryptoviruses a related Partitivirus the *Fusarium poae virus* 1 was analyzed by X-ray crystallography [20,27]. As long as no 3D structure for the alphacryptoviruses is described, it will be difficult to compare these structures of the two genera in a meaningful way. However, it is assumed that they share distinctive features, including a quasi-symmetric CP protein dimerization and formation of a T = 1 capsid structure by 60 dimers by domain swapping [18]. Nevertheless, in this protein interaction study, we are able to find differences between the viruses of two plant infecting genera of the family *Partitiviridae*. We obtained expected CP–RdRp interactions only for the members of the genus *Alphacryptovirus*. The localization of the CP dimers were observed for WCCV-1, RCCV-1 and DCV-1 in the nuclear membrane, whereas the fluorescence signals for the WCCV-2, RCCV-2 and DCV-2 was located in inclusions within the cytoplasm of epidermis cells. CP mutants of WCCV-1 and WCCV-2 showed a different localization of interaction sites in the CP.

From the perspective of the evolutionary relationship, it is interesting to verify protein interactions between viruses in one genus infecting distant host plants and to find no interaction between the type members of genus *Alphacryptovirus* and *Betacryptovirus* in the same host. Together with the different localization of the CP–CP interactions and findings of the CP–RdRp interactions only in the alphacryptoviruses primary indications are given for striking differences in the molecular life cycle of these two virus genera. However, this study is the first protein interaction approach *in planta* for viruses of the family *Partitiviridae* so far and merely one further step to understand the biology of the viruses of the family *Partitiviridae*.

## Appendix

**Table A1:** Oligonucleotides used for amplification of plant cryptic virus ORFs. Virus-specific sequences are shown in lower case at the 3'end; Restriction enzyme recognition sequences/Gibson Assembly sites in upper-case characters.

Virus/Vector	Primer name	Primer sequence
WCCV1	BiFC_WCCV1_CP_s	cgGGATCCatgaatcaagacactcctctcgcc
	BiFC_WCCV1_CP_as	acgcGTCGACttcagcacggttggcagcttg
	BiFC_WCCV1_R_s	gaAGATCTatggattacctaatcactgcatttaaccg
	BiFC_WCCV1_R_as	acgcGTCGACctcgcctggagcattgataaacaa
RCCV1	BiFC_RCCV1_Rs	gaAGATCTatggattacttcatatccgcatttaac
	BiFC_RCCV1_Ras	ccgCTCGAGctcgccaggtgcattgatg
	BiFC_RCCV1_Cs	cgGGATCCatgaatcacaacactcctcctgc
	BiFC_RCCV1_Cas	acgcGTCGACttcagcacggttggcagc
DCV1	BiFC_DCV1CPs	cgGGATCCatggaccccaacgtccctattgc
	BiFC_DCV1CPas	acgcGTCGACttcggcgcggttcgcggcct
	GA12_GA_DCV1Rs	GGATCTGGTGGAGGTGGATCCatggattacctcacaaccgcattc
	GA12_GA_DCV1Ras	GAGGATCGATCCTTAGTCGACctcagcaggatccttaagaaataag
	GA34_GA_DCV1Rs	GAAGGAGATATAACAATGGGATCCatggattacctcacaaccgcattc
	GA34_GA_DCV1Ras	CCAGATCCACCTCCGTCGACctcagcaggatccttaagaaataag
WCCV2	BiFC_WCCV2_R_s	cgGGATCCatgcctcacaactccactcgc
	BiFC_WCCV2_R_as	acgcGTCGACcgggaaatttcttgtggcaggca
	GA12_WCCV2CP_s	GGATCTGGTGGAGGTGGATCCatgtctcctgatgagaaccccac
	GA12_WCCV2CP_as	GAGGATCGATCCTTAGTCGACgacagcggggtaggattcatag
	GA34_WCCV2CP_s	GAAGGAGATATAACAATGGGATCCatgtctcctgatgagaaccccac
	GA34_WCCV2CP_as	CCAGATCCACCTCCGTCGACgacagcggggtaggattcatag
RCCV2	BiFC_RCCV2_R_s	gaAGATCTatgccgttcaactctgctcg
	BiFC_RCCV2_R_as	ACGCgtcgacCGGGAAATTTCTTGTGGCGGG
	BiFC_RCCV2_CP_s	cgGGATCCatgtctactgaagagacccttcct
	BiFC_RCCV2_CP_as	ACGCgtcgacAACAGCGGGGAAGGACTCATA
DCV2	BiFC_DCCV2_Rs	cgGGATCCatgcctttcaactctgttcgcaact
	BiFC_DCCV2_Ras	acgcGTCGACcggaaaactttttgtgctaggcactac
	BiFC_DCCV2_CPs	gaAGATCTatgtcttctacatccctcacatcccg
	BiFC_DCCV2_CPas	acgcGTCGACcacgacggggagagcttcataagg
BiFC 1–2	BiFC_GA_1_2s	GGATCCACCTCCACCAGATCC
	BiFC_GA_1_2as	GTCGACTAAGGATCGATCCTC
BiFC 3–4	BiFC_GA_3_4s	GGATCCCATTGTTATATCTCCTTCG
	BiFC_GA_3_4as	GTCGACGGAGGTGGATCTGG
WCCV1 F1–3	DM_WCCV1CP1as	agcaccgtaaccggtgttgacata
	DM_WCCV1CP2s	gcctacgcacatgacttggatgt
	DM_WCCV1CP3as	cttgtgcatgattaaggagatgtgca
	DM_WCCV1CP4s	tacgctcagtacttcaatggttctgt
WCCV2 F1–3	DM_WCCV2CP91as	ggtagagttaccaggaagtgtagcag
	DM_WCCV2CP02s	agaactgatgtttttcgtgatttgtactca
	DM_WCCV2CP03as	ggtaggcatatgggcactaataacagt
	DM_WCCV2CP04s	gttgtcggtaaggtaattgagtcttttgaac
